# Multimorbidity and Multiple Disabilities: Present Status and the Roles of Rehabilitation

**DOI:** 10.3390/jcm13216351

**Published:** 2024-10-23

**Authors:** Masahiro Kohzuki

**Affiliations:** 1 Department of Health Sciences, Yamagata Prefectural University of Health Sciences, Yamagata 990-2212, Japan; makohzuki@gmail.com; Tel./Fax: +81-23-686-6601; 2 Department of Internal Medicine and Rehabilitation Science, Tohoku University Graduate School of Medicine, Sendai 980-8575, Japan; 3 International Society of Renal Rehabilitation, Sendai 980-8575, Japan

**Keywords:** FITT-VP, multimorbidity, multiple disabilities, rehabilitation, super-aged society

## Abstract

The world is aging, and an increasing number of countries are becoming aged or super-aged societies. Japan has already become the world’s first super-aged society, with an aging rate of 29.1% of the entire population of the country. As of 15 September 2021, there were approximately 36.40 million people aged 65 years and over. The advent of the super-aged society has increased the possibility of multimorbidity and multiple disabilities (MMDs) in the elderly population. According to a survey by the Ministry of Health, Labour and Welfare, the percentage of people with multiple disabilities of all people with physical disabilities has fluctuated between 4.0 and 7.0%, but rapidly increased to 8.9% in 2006 and 17.7% in 2016. This review aimed to establish the present status of MMDs and the rehabilitation for MMDs. In rehabilitation settings, patients with MMDs are more common than patients with a single disease or disability; however, evidence on MMD rehabilitation is insufficient. Inexperienced and unconfident medical professionals are overly cautious in providing rehabilitation; therefore, adequate rehabilitation is not provided. Furthermore, to respond to the rehabilitation needs of patients with MMDs, human resources need to be cultivated, and a scientific basis needs to be built. It is expected that MMD guidelines will soon be developed based on various case studies and surveys. In MMD rehabilitation, it is important to provide “*wider*, *earlier*, *more intimate*, *and more connected rehabilitation*”; for this, the training and cooperation of rehabilitation medical professionals is necessary.

## 1. Introduction

The world is aging, and an increasing number of countries are becoming aged or super-aged societies. The improvement in public hygiene, the advancement in medicine and health care, and the improvement in the standard of living have contributed to the extension of average life expectancy by decreasing infant mortality, the death rate by tuberculosis and infectious diseases, and lifestyle diseases; in particular, the decrease in cerebrovascular diseases has reduced mortality among the middle-aged population [1,2,3].

As the population ages, more people have problems with motor and daily living functions due to sarcopenia, frailty, decreased balance function, and malnutrition [4,5,6]. In addition, with age, the incidence of cardiovascular diseases such as heart failure, myocardial infarction, and cerebrovascular disease, respiratory diseases such as COPD, dementia, cancer, musculoskeletal diseases, and kidney diseases such as chronic kidney disease also increases, accelerating the decline in motor and daily living functions [7,8,9,10,11,12]. The advent of the super-aged society has increased the possibility of multimorbidity and multiple disabilities in the elderly population.

Moreover, the global prevalence of activity limitation is substantially higher in women than in men and in low-income and middle-income countries than in high-income countries [2]. Strategies are needed to prevent and mitigate activity limitations globally, with particular emphasis on low-income countries and women [13].

Japan has become one of the world’s leading aging societies, with an aging rate of 29.1% of the entire population of the country. As of 15 September 2021, there were approximately 36.40 million people aged 65 years and over [14], and the number of people with multiple disabilities caused by multiple diseases is increasing. According to a survey by the Ministry of Health, Labour and Welfare, the percentage of people with multiple disabilities of all people with physical disabilities has fluctuated between 4.0 and 7.0%, but rapidly increased to 8.9% in 2006 and 17.7% in 2016 [15]. This number of people with multiple disabilities refers to the number of people with two or more physical disabilities (visual impairment, hearing/speech impairment, limb disability, and visceral disability), and if intellectual disabilities and mental disabilities are also included, the number of people with multiple disabilities is estimated to be even higher.

This review aimed to establish the present status of MMDs and the rehabilitation for MMDs.

## 2. Multimorbidity and Comorbidity

The English synonyms of multiple or overlapping diseases are “multimorbidity” or “comorbidity.” Multimorbidity is the simultaneous occurrence of two or more diseases, and comorbidity is not simply a combination of diseases but a combination of closely related diseases [16]. For example, comorbidities can be divided into three clusters: (1) hypertension and diabetes; (2) asthma, bronchitis, arthritis, osteoporosis, and depression; and (3) cancer, heart disease, and stroke [17].

Patients with multiple or overlapping diseases experience difficulties in daily life, more frequent hospitalizations, longer hospital stays, a lower quality of life, heavier mental burden, more postoperative complications, and higher medical costs [18]. In the field of rehabilitation, the problem is not hypertension or diabetes in patients with multiple or overlapping diseases, but rather stroke, heart disease, renal failure, visual impairment, and sensory impairment resulting from organ damage caused by hypertension and diabetes. Therefore, the terminology of multiple diseases and comorbid diseases is not useful in rehabilitation, and a different approach is required [18].

## 3. Definition of Multimorbidity and Multiple Disabilities (MMDs)

Therefore, there is a need for a new term to describe the overlapping conditions of disabilities, regardless of age or sex, as seen in rehabilitation in general, and the term “multimorbidity and multiple disabilities (MMD)” was proposed in 2015 [18,19]. Furthermore, MMD is defined as “an overlapping condition of two or more of the following: visual impairment, hearing or balance disorder, speech/language or masticatory disorder, physical disability, visceral disability, intellectual disability, mental disability, and higher brain dysfunction; or an overlapping condition of two or more of the following seven visceral disorders: cardiac dysfunction, renal dysfunction, hepatic dysfunction, respiratory dysfunction, bladder/rectal dysfunction, small intestinal dysfunction, and immune dysfunction due to human immunodeficiency virus” [18,19]. Ten years have passed since then, and the term MMD has come to be used without any particular objection, both in Japan and overseas.

The original English translation of the overlapping conditions of disabilities should be “multiple disability.” In the pediatric field, however, “multiple disability” had already been used synonymously with “severe intellectual disability and cerebral palsy” until 2013. Therefore, the English translation of overlapping conditions of disabilities was named “multimorbidity and multiple disabilities (MMD)” in 2015 [18,19].

In the statutory disability classification of MMDs in Japan, the most common combination of disabilities in patients with MMDs is physical disability and visceral disability. In Japan, 17.7% of the patients with MMDs have two or more disabilities [15]. Meanwhile, the number of patients with MMDs is even higher in the real world, and 32–62% of stroke patients in the United States have ischemic heart disease [20]. Furthermore, 33% of elderly patients with heart failure also have chronic obstructive pulmonary disease (COPD) [21], and 25% of elderly patients with COPD also have heart failure [22]. In Japan, the proportion of patients with chronic kidney disease (CKD) is approximately 30% in their 70s and approximately 50% in those aged 80 years or older [23], and many patients with MMDs also have renal dysfunction.

## 4. Definition of MMD Rehabilitation

Patients with MMDs often have reduced exercise tolerance, activities of daily living (ADL), and quality of life (QOL); it goes without saying that rehabilitation to improve these is important. In 2015, MMD rehabilitation was defined as “a long-term comprehensive program including health checks, addressing the relationship between organs and diseases, exercise therapy, diet and fluid management, drug therapy, education, and mental and psychological support, with the aim of reducing the physical and mental impact of overlapping disabilities caused by multiple diseases, controlling symptoms, improving life prognosis, and improving psychosocial and occupational conditions” [18,19].

## 5. Advantages and Disadvantages of MMD Rehabilitation

MMD rehabilitation has the following advantages and disadvantages.

### 5.1. Disadvantage 1: Excessive Strain on Organs

Upper limb walking significantly increases oxygen consumption and heart rate compared to walking at the same speed, increasing the strain on the heart and lungs. In other words, hemiplegic patients with cerebrovascular disease are at risk of being susceptible to angina and heart failure. In patients with stroke, the presence of heart disease hinders the original purpose of stroke rehabilitation [24,25,26].

### 5.2. Disadvantage 2: Many Things to Keep in Mind During Rehabilitation

Accidents that can occur during the rehabilitation of patients with MMDs include a loss of consciousness, chest pain, dyspnea, dizziness, arrhythmia, falls, and fractures. Therefore, there are many things to keep in mind during rehabilitation, and sufficient supervision and personnel are required. When increasing the load, it is necessary to check for the appearance of such symptoms, changes in the electrocardiogram, and the presence or absence of a decrease in SpO2. In addition, in patients with heart failure, it is advisable to regularly monitor changes in weight and the presence or absence of edema, and to regularly measure blood brain natriuretic peptide (BNP) levels [24].

### 5.3. Advantage 1: Greater Rehabilitation Effects Can Be Expected

Regarding the overlap of cardiac and respiratory dysfunction, a randomized controlled trial targeting elderly patients with chronic obstructive pulmonary disease (COPD) and heart failure showed that 4 months of rehabilitation significantly improved exercise tolerance and quality of life and reduced the number of rehospitalizations and deaths compared to the control group [27]. Furthermore, it has been reported that the rehabilitation effect in heart failure patients with COPD is equivalent to that in heart failure patients without COPD [28]; therefore, rehabilitation should not be discontinued simply because the patient has MMDs.

Patients with MMDs spend a lot of time in bed, which can lead to a disuse syndrome of various organs [29]; moreover, a low-activity lifestyle itself is a risk factor for the development of diseases and disabilities. In general, the lower the physical strength, the greater the effect of rehabilitation; therefore, patients with MMDs who have not received sufficient rehabilitation are more likely to benefit from rehabilitation [30]. We experienced a case in which a patient waiting for a lung transplant was able to temporarily avoid lung transplantation by performing whole-body endurance training focusing on the lower limbs [31]. This is a classic example of how rehabilitation is extremely effective in improving respiratory impairment and severe frailty.

### 5.4. Advantage 2: Extended Lifespan

The goal of stroke rehabilitation is often to return to work or home, but the goal of visceral disease rehabilitation (cardiac, pulmonary, and renal rehabilitation) is not limited to that; it also aims at extending the lifespan. In other words, by working towards the rehabilitation goal of MMDs rather than simply returning to work or home, one can also expect to extend one’s lifespan [1].

## 6. Recent Topics in MMD Rehabilitation

### 6.1. Characteristics of Patients with MMDs and Key Points for Rehabilitation

Table 1 shows the characteristics of patients with MMDs and key points for rehabilitation [24]. Because the treatment of one disease can easily affect other diseases, acquiring sufficient knowledge about organ disorders and considering organ connections and the overall condition when providing treatment is essential. As pathological conditions are diverse and there are large individual differences, tailor-made treatments, including psychological and environmental aspects, are necessary. Because immobility can easily cause complications (disuse syndrome), rehabilitation treatment should be initiated early, and efforts should be made to continue it.

### 6.2. One Day of Complete Rest Will Age One Two Years

After the age of 20, humans lose muscle mass, strength, and endurance by approximately 1% each year. If one day is spent at complete rest, muscle mass and strength decrease by approximately 2%, mainly in the antigravity muscles of the trunk and lower limbs. In the 1966 Dallas Bed Rest and Training Study, five 20-year-old men underwent 3 weeks of bed rest and 8 weeks of heavy endurance training to test the limits of the cardiovascular system [32]. In the original five participants of the Dallas Bed Rest and Training Study, maximum oxygen uptake (VO_2_max) declined after 40 years of age because of a balanced decrease in the central and peripheral determinants of oxygen uptake. The rate of decline in VO_2_max and its components accelerated after the age of 50 years secondary to age and clinical comorbidities. The net proportional decline in VO_2_max over a period of 40 years was comparable to that experienced after 3 weeks of strict bed rest at the age of 20 years (27% vs. 26%, respectively) [33]. In other words, one day of complete rest will age one two years [34].

**Figure 1:**
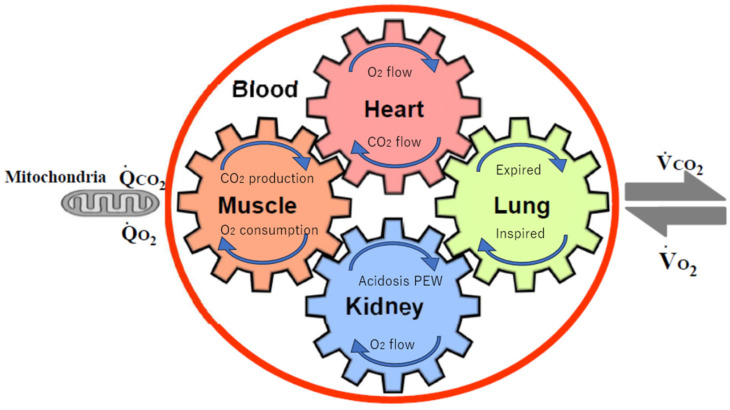
The five major determinants of VO_2_max, peak VO_2_, and their relationships (adapted from [30]). The gears represent the functional interdependence of the physiological components of the system. Cardiac output, pulmonary diffusion capacity, oxygen carrying capacity, renal function, metabolic acidosis, and other peripheral limitations, such as muscle diffusion capacity, mitochondrial enzymes, and capillary density, are all examples of VO_2_max determinants. VO_2_, O_2_ uptake; VCO_2_, CO_2_ output; QCO_2_, CO_2_ production; QO_2_, O_2_ consumption by cells.

Endurance is expressed as VO_2_max and was previously determined by four factors: the heart, lungs, muscles, and blood; however, it has recently been determined by five factors, including the kidneys (Figure 1) [30]. Renal dysfunction specifically progresses to sarcopenia and frailty due to metabolic acidosis and protein energy wasting [29]. In other words, multiple disorders, including kidney dysfunction, accelerate the decline in endurance. For a patient undergoing dialysis, a 4 h rest dialysis 3 times a week for 1 year (52 weeks) means that the patient will be bedridden for an extra 26 days (4 h × 3 × 52 ÷ 24 = 26 days) per year, and the dialysis treatment itself increases the risk of developing disuse syndromes such as frailty and sarcopenia. In other words, exercise therapy is important for preventing and treating frailty, sarcopenia, and disuse syndrome in patients undergoing dialysis.

We conducted a randomized controlled trial to investigate whether the effect of 40 min of ergometer exercise during dialysis for 12 weeks differed depending on the equipment [35]. Quadriceps muscle strength was found to increase significantly when equipment capable of accurately applying a load up to a Borg score of 13 (Terrace Ergo™) was used, whereas muscle strength decreased significantly when equipment unable to accurately apply a load up to a Borg score of 13 was used (Table 2) [35].

### 6.3. FITT-VP: New Rehabilitation Prescriptions

**Figure 2:**
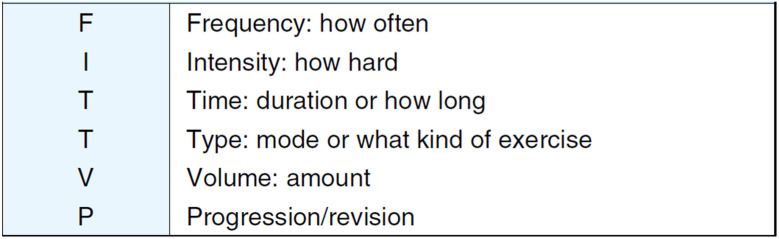
FITT-VP Principles of Exercise Prescription (adapted from Ref. [36]). Rehabilitation prescriptions have been determined based on the FITT-VP, which adds exercise volume (V) and incremental increase/correction (P) to the FITT (frequency (F), intensity (I), time (T), and type (T)). V is the product of “FIT,” i.e., the frequency, intensity, and duration of exercise.

Traditional rehabilitation prescriptions are based on FITT: frequency (F), intensity (I), time (T), and type (T). However, although it is best to start with the lightest load for each item, there was a tendency to stop there, and some facilities did not achieve sufficient rehabilitation effects due to this. Therefore, in recent years, rehabilitation prescriptions have been determined based on the FITT-VP, which adds exercise volume (V) and incremental increase/correction (P) to the FITT (Figure 2) [36]. In particular, exercise volume is the product of “FIT,” i.e., the frequency, intensity, and duration of exercise, and the exercise volume in exercise prescriptions should be gradually increased and periodically corrected [36]. In other words, exercise therapy with a low exercise volume (V = F × I × T) required for “FIT” during dialysis is ineffective, and it is necessary to perform exercise therapy with a sufficient amount of exercise (V) within a safe range.

### 6.4. Effects of MMD Rehabilitation on Patients with Heart and Kidney Disabilities

Regarding the overlap of cardiac and renal dysfunction, the coexistence of renal dysfunction in patients with myocardial infarction (MI) increases the subsequent total mortality and cardiovascular-related mortality. However, it has been reported that CKD dialysis patients who underwent coronary artery bypass surgery experienced a reduction in both total mortality and cardiac mortality by more than 30% after undergoing rehabilitation [37]. In patients with heart failure, mortality and rehospitalization rates are closely related to renal function; however, mortality is also affected by exercise tolerance (maximum oxygen intake) [38].

**Figure 3:**
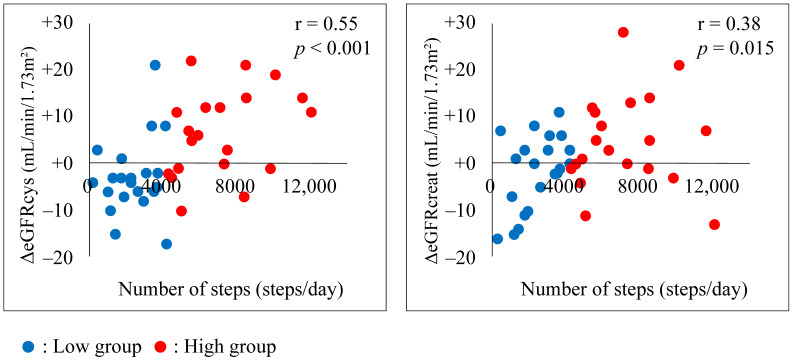
Association between the number of steps and ΔeGFRcys or ΔeGFRcreat (adapted from [40]). The association between the number of steps taken and eGFRcys in all patients is shown. Pearson’s correlation analysis revealed significant correlations between the number of steps and both eGFR parameters, with a higher correlation between ΔeGFRcys and the number of steps than between ΔeGFRcreat and the number of steps. eGFRcreat, creatine-based estimated glomerular filtration rate; eGFRcys, cystatin-based estimated glomerular filtration rate.

In addition, patients with conservative CKD who develop MI experience improvements in renal function (eGFR) after receiving convalescent cardiac rehabilitation [39]. In our intervention study, we transitioned patients with MI to home convalescent myocardial rehabilitation after MI and found that maintaining a high number of steps per day at home also led to the suppression of renal function decline (Figure 3) [40].

### 6.5. Renal Rehabilitation: One Representative of MMDs Is Developing Rapidly

Regarding renal rehabilitation, milestones include the establishment of the Japanese Society of Renal Rehabilitation (JSRR) in 2011, the certification of renal rehabilitation instructors in 2018, renal rehabilitation guidelines (Japanese version in 2018 [30,41], English version in 2019 [42]), and the establishment of the International Society of Renal Rehabilitation (ISRR) in 2020 [30]. In the 2022 revision of medical treatment fees in Japan, the scope of renal rehabilitation was expanded to include dialysis patients who became eligible for the exercise instruction surcharge during dialysis. From the establishment of the society to the calculation of medical treatment fees, all of these were world-first efforts, and it is no exaggeration to say that the JSRR is leading the world in renal rehabilitation [30,43]. At the first annual scientific meeting of the JSRR, there were 80 members. Although it was a new initiative, it has since developed smoothly, with the number of members increasing to 3300 in April 2024; more than 9000 people attended the three Renal Rehabilitation Guideline Seminars held by the JSRR in 2022, 2023, and 2024 [30].

### 6.6. Expectations for MMD Rehabilitation: The Watchword Is “Wider, Faster, More Intensive, Connected Rehabilitation”

Many of the rehabilitation guidelines to date have, in principle, targeted a single disease or disability. Therefore, evidence has also been limited to a single disease or disability, and evidence for MMD rehabilitation has not been presented sufficiently [19,44]. However, in the real world, there are more patients with MMDs than with a single disease or disability.

Rehabilitation plans must be made and implemented even in situations not specified in the guidelines, which has caused confusion regarding the implementation of MMD rehabilitation. Inexperienced or unconfident medical professionals tend to be too cautious in providing rehabilitation and provide insufficient rehabilitation. “I go to the hospital for rehabilitation, but I can’t do it properly” is a major complaint of patients and their families, and it is desirable to create guidelines and manuals as soon as possible.

In MMD rehabilitation, it is important to provide “rehabilitation that is more extensive (do not give up even if there are multiple disabilities), earlier (start early), more intensive (increase treatment time and increase independent training in bed or at home), and connected (connecting from acute wards to recovery wards, and then to the living and maintenance phases)” [45].

To achieve this, medical rehabilitation professionals must cooperate with each other. Rehabilitation medical professionals must study the pathophysiology of cardiovascular, renal, respiratory, and metabolic diseases, the connections and disorders of organs, electrocardiograms, respiratory function tests, and blood gas data, so that they can respond flexibly to exercise and dietary therapy for multiple disorders.

As lifestyle and maintenance phase rehabilitation is mainly performed at home, it is important that the rehabilitation menu be performed at home and that patients and their families continue without anxiety. The success or failure of this depends on the guidance and awareness of medical institutions, visiting rehabilitation, and day rehabilitation.

This review covers the definition of MMD and the results of rehabilitation studies on MMDs. However, the limitations of this review are that it does not cover all quantitative and qualitative research findings on MMDs, and it did not search for research studies on MMD patients themselves and their families/caregivers. These points need to be thoroughly considered in the future.

## 7. Conclusions

In the rehabilitation of patients with MMDs, it is essential to consider the individual differences and wishes of each patient in terms of their physical, mental, psychological, and social backgrounds, set individual treatment goals, and provide comprehensive medical care. Simultaneously, it is necessary to consider the problems specific to each organ and the connections between the brain, heart, lungs, bones, and joints. In Japan, the world’s largest super-aging society, there are many patients with MMDs. However, there are few appropriate MMD rehabilitation intervention studies, and the evidence for MMD rehabilitation is insufficient. This is an extremely serious problem. Future large RCTs should focus more on the effects of MMD rehabilitation and exercise programs, as these topics and exercise types have not yet been studied. To improve the functional and life prognoses of patients with MMDs, it is necessary to review the conventional FITT for organ-specific rehabilitation and conduct sufficient verification in the future.

Furthermore, to respond to the rehabilitation needs of patients with MMDs, human resources need to be cultivated, and a scientific basis needs to be built. It is expected that MMD guidelines will soon be developed. In MMD rehabilitation, it is important to provide “wider, earlier, more intimate, and more connected rehabilitation”. To achieve this, medical rehabilitation professionals must cooperate with each other.

## Figures and Tables

**Table 1:** Key points for MMD rehabilitation (modified from Ref. [24]).

**Rehabilitation principles**
(i) The treatment of one disease can easily affect other diseases; acquire sufficient knowledge about organ disorders, and consider organ connections and the overall condition when providing treatment.
(ii) Since pathological conditions are diverse and there are large individual differences, tailor-made treatments, including psychological and environmental aspects, are necessary.
(iii) Because immobility can easily cause complications (disuse syndrome), rehabilitation treatment should be initiated early, and efforts should be made to continue it.
**Rehabilitation diagnosis**
(i) It is not uncommon for patients to have severe disease but no clear clinical symptoms. A physician should not be overconfident about the absence of subjective symptoms.
(ii) A physician should use weight, blood pressure, pulse rate, oxygen saturation, electrocardiograms, blood biochemistry test results, and urine test results.
(iii) A physician should perform rigorous exercise stress tests.
**Setting goals for rehabilitation treatment**
(i) It is necessary to give more consideration to the QOL when providing treatment.
(ii) Listen carefully to the patient’s current lifestyle and what gives them meaning in life and set treatment goals while always keeping in mind the gap between what is right and what can be achieved.
(iii) Create an individual program that incorporates the idea of “Adding Life to Years” as well as “Adding Life to Years and Years to Life”.
(iv) A complete cure of the disease is often impossible, and the issue is how to return the patient to their home and society.
(v) Once a certain degree of improvement in ADL is observed during hospitalization, a system is created to continue rehabilitation treatment at home.
**Rehabilitation treatment**
(i) Low- to moderate-intensity exercise is used rather than vigorous exercise, and the time and frequency are gradually increased.
(ii) As complications not directly related to the original disease are likely to occur, exercise therapy should take longer for warm-ups and cool-downs, and time is taken to progress in the intensity of exercise.
(iii) Close attention should be paid to changes in medications and meal menus, nutritional status, and the presence or absence of dehydration.
(iv) In cases of cognitive decline, hearing impairment, or visual impairment, responses should be loud, clear, slow, and careful, and creative teaching materials should be used to ensure that the training is easy to understand.
(v) Ensure that patients and their families understand the training content.
**Rehabilitation support**
(i) Assemble a multidisciplinary rehabilitation team to support effective, comprehensive, and integrated intervention planning in rehabilitation programs.
(ii) Consider the use of telerehabilitation services.
(iii) Adjust the environment and utilize social resources to support patients’ social activities.

**Table 2:** Changes after 12 weeks of intradialytic exercise with traditional or electric exercise bikes (quoted from Ref. [35]). Quadriceps muscle strength increased significantly when the equipment was capable of accurately applying a load up to a Borg score of 13 (Tex group), whereas muscle strength decreased significantly when the equipment (Elex group) was unable to accurately apply a load up to a Borg score of 13.

	Tex Group (*n* = 8)		Elex Group (*n* = 7)		Interaction *p* Value	Intergroup Comparison *p* Value		The Effect of Exercise *p* Value	
	Pre	Post	Pre	Post		Pre	Post	Tex	Elex
DW (kg)	52.4 ± 2.8	52.4 ± 2.5	51.3 ± 5.4	51.4 ± 2.6	n.s.	n.s.	n.s.	n.s.	n.s.
Kt/V (mL/min)	1.7 ± 0.1	1.6 ± 0.2	1.6 ± 0.2	1.6 ± 0.1	n.s.	n.s.	n.s.	n.s.	n.s.
TP (g/dL)	6.8 ± 0.2	6.8 ± 0.3	6.7 ± 0.2	6.8 ± 0.1	n.s.	n.s.	n.s.	n.s.	n.s.
Hb (g/dL)	10.8 ± 0.2	10.8 ± 0.2	11.3 ± 0.5	11.0 ± 0.3	n.s.	n.s.	n.s.	n.s.	n.s.
Non-HDL-C (mg/dL)	123.4 ± 7.0	123.4 ± 6.6	124.6 ± 16.9	128.3 ± 7.8	n.s.	n.s.	n.s.	n.s.	n.s.
MPQ (kgf)	26.2 ± 1.5	28.1 ± 1.6	27.6 ± 1.5	19.3 ± 2.8	0.025	n.s.	0.042	0.031	0.046.
CS-30 (no. of times)	12.8 ± 0.9	14.8 ± 0.7	11.7 ± 2.4	14.4 ± 2.3	n.s.	n.s.	n.s.	n.s.	n.s.
TUG (s)	7.5 ± 0.6	6.3 ± 0.3	7.9 ± 1.1	8.9 ± 1.8	n.s.	n.s.	n.s.	n.s.	n.s.
6MWD (m)	479.5 ± 34.4	549.5 ± 34.7	389.1 ± 75.8	390.6 ± 79.2	n.s.	0.049	0.048	0.028	n.s.

Values are expressed as mean ± standard deviation. n.s.: not significant, pre: pre-intervention, post: post-intervention, DW: dry weight, Kt/V: HD efficiency, TP: total protein, Hb: hemoglobin, non-HDL-C: non-high- density lipoprotein cholesterol, MPQ: muscular power of the quadriceps, CS-30: 30-s chair stand test, TUG: time up and go test, 6MWD: 6-min walking distance test.

## Data Availability

Not applicable.
